# Bile acids in Alzheimer’s disease: a double-edged sword in gut–liver–brain signaling and neurodegeneration

**DOI:** 10.1007/s00210-026-05516-1

**Published:** 2026-06-09

**Authors:** Rehana Basri, Hayder M. Al-kuraishy, Mubarak Alruwaili, Ali I. Al-Gareeb, Ali K. Albuhadily, Athanasios Alexiou, Marios Papadakis, Safaa A. Faheem, Gaber El-Saber Batiha

**Affiliations:** 1 Department of Internal Medicine, College of Medicine, Jouf University, Sakaka, 72345 Aljouf Saudi Arabia; 2 Department of Clinical Pharmacology and Medicine, College of Medicine, Mustansiriyah University, Baghdad, Iraq; 3 Department of Internal Medicine, College of Medicine, Jouf University, Sakaka, Saudi Arabia; 4 Department of Clinical Pharmacology and Medicine, Jabir Ibn Hayyan Medical University, Al-Ameer Qu./Najaf, P.O. Box (13), Kufa, Iraq; 5 European Academy of Sciences and Arts, Vienna, Austria; 6 University Centre for Research & Development, Chandigarh University, Chandigarh-Ludhiana Highway, Mohali, Punjab India; 7 Faculty of Medicine, Health Sciences School, University of Ioannina, University Campus, 45110 Ioannina, Greece; 8 Department of Pharmacology and Toxicology, Faculty of Pharmacy, Egyptian Russian University, Cairo-Suez Road, Badr City, 11829 Cairo Egypt; 9 Department of Pharmacology and Therapeutics, Faculty of Veterinary Medicine, Damanhour University, Damanhour, 22511 AlBeheira Egypt

**Keywords:** Bile acids, Neuroprotective effects, Alzheimer’s disease, Glucagon-like peptide 1, Fibroblast growth factor 19, Farnesoid X receptor, Takeda G protein-coupled receptor 5, G-protein-coupled bile acid receptor 1

## Abstract

Alzheimer’s disease (AD) is a progressive neurodegenerative disorder characterized by amyloid-β (Aβ) deposition, tau pathology, synaptic dysfunction, neuroinflammation, and metabolic impairment. Increasing evidence suggests that bile acids, traditionally recognized for their roles in lipid digestion and hepatic metabolism, act as endocrine signaling molecules that influence central nervous system (CNS) homeostasis. Through enterohepatic circulation and microbiota-dependent biotransformation, bile acid composition is dynamically regulated and can modulate peripheral metabolic and immune pathways with downstream effects on the brain. Notably, bile acid signaling via key receptors such as the farnesoid X receptor (FXR) and the Takeda G protein-coupled receptor 5 and G-protein-coupled bile acid receptor 1 (TGR5/GPBAR1) has emerged as a mechanistic bridge linking liver–gut physiology to neuroinflammatory and neurodegenerative processes. Altered bile acid profiles have been reported in AD and mild cognitive impairment, with accumulating findings suggesting that hydrophobic secondary bile acids may contribute to blood–brain barrier (BBB) disruption and neurotoxicity. In contrast, hydrophilic bile acids may exert neuroprotective and anti-inflammatory effects. In addition, bile acids drive the release of gut hormones such as glucagon-like peptide 1 (GLP-1) and fibroblast growth factor 19 (FGF19), highlighting indirect neurometabolic pathways relevant to cognition and neurodegeneration. This narrative review synthesizes current biochemical, experimental, and clinical evidence supporting a role for bile acid signaling in AD pathogenesis and progression. We discuss receptor-mediated pathways, microbiota-bile acid interactions, neuroimmune modulation, and translational perspectives, proposing that bile acid–based biomarkers and therapeutic strategies targeting FXR/TGR5 signaling may represent promising avenues for future AD intervention.

## Introduction

**Fig. 1:**
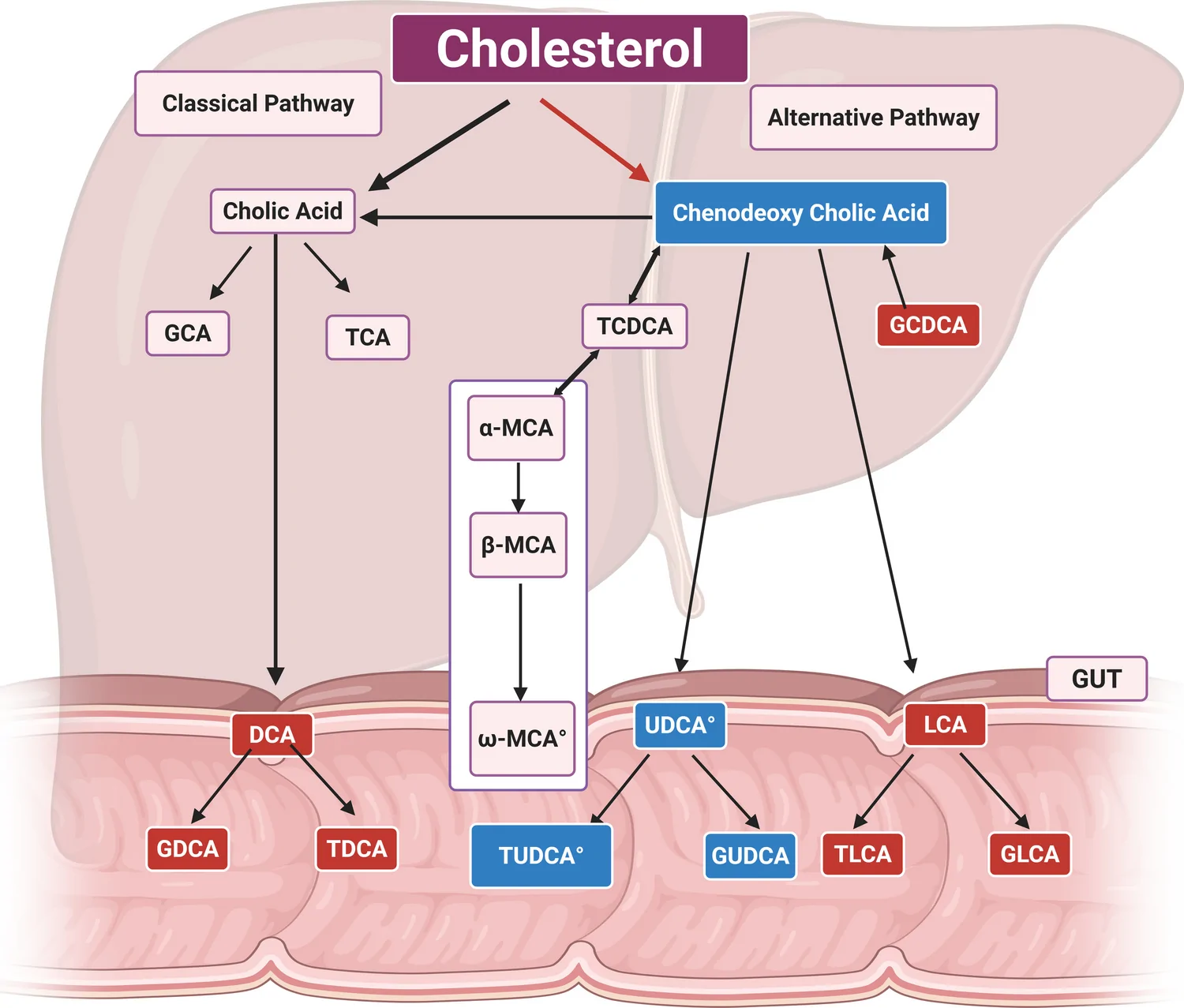
Hepatic–intestinal bile acid biosynthesis and microbial biotransformation from cholesterol (liver–gut axis). Cholesterol is converted in hepatocytes into the primary bile acids cholic acid (CA), predominantly via the classical pathway, and chenodeoxycholic acid (CDCA), primarily via the alternative pathway. Primary bile acids undergo conjugation with glycine (G-) or taurine (T-), generating bile salts including GCA, TCA, and GCDCA. Following secretion into the intestine, gut microbiota mediate deconjugation and 7α-dehydroxylation to form the secondary bile acids deoxycholic acid (DCA) (from CA) and lithocholic acid (LCA) (from CDCA), which may subsequently be reconjugated (e.g., GDCA/TDCA and GLCA/TLCA). Ursodeoxycholic acid (UDCA) and tauroursodeoxycholic acid (TUDCA) represent hydrophilic secondary bile acids that participate in enterohepatic cycling. In rodents, chenodeoxycholic acid can be further converted into muricholic acids (α-MCA, β-MCA, and ω-MCA), which are rodent-specific bile acids with distinct FXR-modulating properties. Color coding distinguishes primary bile acids, secondary bile acids, and their conjugated derivatives. Arrows indicate the principal metabolic conversions and enterohepatic circulation between the liver and intestine

Bile acids are amphipathic small molecules for digestion and enhance the absorption of fat-soluble vitamins and dietary lipids (Aaldijk and Vermeiren 2022). Bile acids are synthesized by hepatocytes in the liver, stored in the gallbladder, and play a crucial role in regulating cholesterol homeostasis and gastrointestinal function (Aaldijk et al. 2023). The released bile acids from the liver into the intestine are called primary bile acids, such as chenodeoxycholic acid and cholic acid, that undergo modifications and reactions by gut bacteria and enterohepatic circulation to form secondary bile acids, such as ursodeoxycholic acid, lithocholic acid, and deoxycholic acid (Al-Gareeb et al. 2025). Under normal physiological conditions, approximately 95% of bile acids are efficiently recycled through the enterohepatic circulation and stored in the gallbladder. In comparison, only about 5% escape intestinal reabsorption and are excreted in the feces (Al-Gareeb et al. 2025). This loss is compensated by the continuous de novo synthesis of primary bile acids in the liver (Al-Kuraishy et al. 2024). The increased amount of formed bile acids inhibits the production of hepatic bile acids through a negative feedback mechanism to preserve bile acid homeostasis (Al-Kuraishy et al. 2024). Bile acids are predominantly produced from hydroxycholesterol by classical and alternative pathways (Al-Kuraishy et al. 2024). To provide an overview of bile acid biosynthesis and metabolism, Fig. 1 schematically illustrates the classical and alternative pathways of bile acid synthesis, highlighting the formation of primary bile acids from cholesterol and their subsequent conversion into secondary bile acids by gut microbiota. Hydroxycholesterol, which is also found in other organs, such as the brain and lungs, can be synthesized from bile acids (Al-Kuraishy et al. 2024). Approximately 500 mg of cholesterol per day is utilized for bile acid synthesis under normal physiological conditions. Bile acids play a protective role against cholesterol gallstone formation by maintaining cholesterol solubility; cholesterol gallstones arise when cholesterol secretion exceeds bile acid solubilizing capacity or when bile acid production is reduced (Al-Kuraishy et al. 2025).

**Fig. 2:**
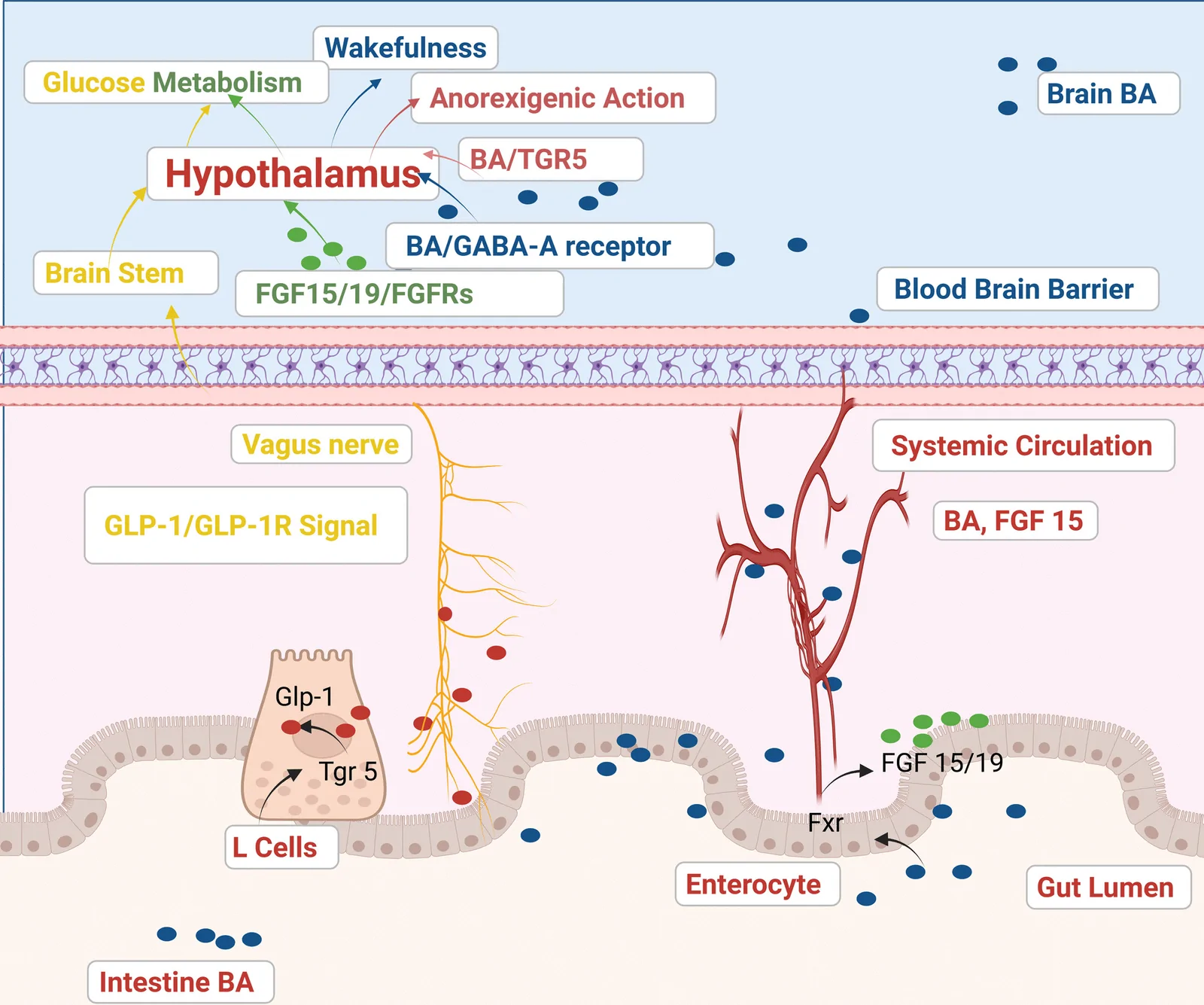
Central actions of bile acids (BAs) within the liver–gut–brain axis. Intestinal bile acids (blue dots) activate Takeda G-protein-coupled receptor 5 (TGR5) on enteroendocrine L cells, stimulating GLP-1 release and downstream GLP-1/GLP-1R signaling, which is transmitted to the brain via vagal afferent pathways. In parallel, bile acids activate intestinal FXR in enterocytes, inducing secretion of FGF15/19, which enters the systemic circulation and signals centrally through FGF15/19–FGFRs in hypothalamic networks. Circulating bile acids and FGF15/19 may reach the brain through the blood–brain barrier (BBB) (via passive diffusion for lipophilic species and/or transporter-mediated uptake for hydrophilic species), contributing to local “brain BA” pools. Within the CNS, particularly the hypothalamus and brainstem, bile acids modulate neurobehavioral and metabolic endpoints—including wakefulness, anorexigenic (satiety) responses, and glucose metabolism—through activation of bile acid receptors such as TGR5 and interaction with neurotransmitter systems (including GABA-A receptor–linked signaling). Color coding distinguishes functional domains within the liver–gut–brain axis: Central nervous system structures and signaling pathways are shown in blue tones; gut-derived mediators and enteroendocrine signaling components are shown in yellow/green tones; and systemic bile acid–dependent endocrine pathways are indicated in red. This color scheme visually separates central, peripheral, and bile acid–mediated mechanisms

Furthermore, bile acids serve as signaling molecules that facilitate crosstalk between the liver, gut, and brain, thereby forming a liver–gut–brain axis (Al-Kuraishy et al. 2025, 2025). The liver, gut, and the brain are interrelated primarily through systemic circulation and the vagus nerve. Hence, bile acids can cross the intestinal barrier into the systemic circulation and, through the blood–brain barrier (BBB), can reach the brain (Al-Kuraishy 2025). Notably, the source of conjugated and unconjugated bile acids in the brain remains unclear (Al-Kuraishy et al. 2025) (Fig.2). Nevertheless, primary bile acids are de novo synthesized by the brain and are involved in the metabolism of brain cholesterol (Alqahtani et al. 2025). However, most of the hydrophilic brain bile acids are derived from systemic circulation, where lipophilic bile acids can directly pass through the BBB. The hydrophilic bile acids cross the BBB through bile acid transporters (Alsaleem et al. 2024). Various bile acid transporters are expressed in the CNS, including sodium-taurocholate co-transporting polypeptide (NTCP), bile salt export pump (BSEP), and apical sodium-dependent bile acid transporter (ASBT) in the hypothalamus and other brain regions (Alsaleem et al. 2024, 2025). Recently, it has been reported that several endogenous bile acids can rapidly reach the hypothalamus and transiently increase within 30 to 60 min after physiological feeding at the beginning of the dark phase in mice (Alsaleem et al. 2024, 2025). Nearly 40 diverse primary and secondary bile acids are present in the brain. In particular, enzymes essential for the synthesis of primary bile acids are expressed in astrocytes and oligodendrocytes (Baloni et al. 2020). However, the enzymes required for the synthesis of secondary bile acids are not detected in the brain (Alsaleem et al. 2024), suggesting that secondary bile acids in the brain are derived from the systemic circulation. Notably, unconjugated bile acids are small, lipid-soluble molecules that can cross the BBB (Alsaleem et al. 2024). Therefore, brain concentration of unconjugated bile acids positively correlates with their plasma levels. On the contrary, conjugated bile acids are large, water-soluble molecules that cannot cross the BBB; thus, they depend on bile acid transporters, and their brain concentrations are not correlated with their plasma levels; therefore, conjugated bile acids have limited CNS effects (Al-Kuraishy et al. 2025).

Importantly, bile acids act centrally by stimulating numerous receptors, such as the brain farnesoid X receptor (FXR) and the Takeda G protein-coupled receptor 5 (TGR5), which regulate neurotransmitter activity and related neurobehavioral responses (Bazzari et al. 2019). It has been demonstrated that bile acids, through the activation of these receptors, regulate memory and cognitive functions (Belka et al. 2024). Primarily, TGR5, which is widely expressed in neurons and astrocytes, constrains microglial activation and neuroinflammation (Belka et al. 2024). Also, FXR negatively regulates the transcription of proinflammatory cytokines and inflammatory molecules (Bhargava et al. 2020). Therefore, the TGR5/FXR axis may have neuroprotective effects against neuroinflammation in different neurodegenerative diseases, including Alzheimer’s disease (AD). Significantly, bile acids affect brain function either directly via activation of FXR and TGR5 (Bazzari et al. 2019) or indirectly through modulation of bile acid receptors in the gut that induce the expression of the neuroprotective glucagon-like peptide 1 (GLP-1) and fibroblast growth factor 19 (FGF19) (Bazzari et al. 2019). Moreover, bile acids regulate glucose homeostasis and brain insulin signaling (Brighton et al. 2015). These findings highlighted diverse effects of bile acids on the brain (Table1). Different studies highlighted that alteration of bile acids is linked with the development of cognitive impairment and AD. Findings from experimental and clinical studies indicated that bile acids have neuroprotective anti-inflammatory effects against AD development (Collins et al. 2023; D’Anniballe De Salles et al. 2026). Despite these findings, the exact molecular mechanisms by which bile acids contribute to AD remain unclear. Therefore, this review aims to discuss and explain the underlying molecular mechanisms of bile acids in AD.

**Table 1:** Effects of bile acids on the brain

Effects of bile acids on the brain	Ref
Bile acids serve as signaling molecules that facilitate crosstalk between the liver, gut, and brain, thereby forming a liver–gut–brain axis	(Al-Kuraishy et al. 2025, 2025)
The brain de novo synthesizes the primary bile acids* and* is involved in the metabolism of brain cholesterol	(Alqahtani et al. 2025)
The hydrophilic brain bile acids are derived from systemic circulation, where lipophilic bile acids can directly pass through the BBB. The hydrophilic bile acids cross the BBB through bile acid transporters, including NTCP, BSEP, and ASBT, in the hypothalamus and other brain regions	(Alsaleem et al. 2024)
The enzymes essential for the synthesis of primary bile acids are expressed in astrocytes and oligodendrocytes. However, the enzymes required for the synthesis of secondary bile acids are not detected in the brain	(Alsaleem et al. 2024; Baloni et al. 2020)
The brain concentration of unconjugated bile acids is positively correlated with their plasma levels. However, brain concentrations of the conjugated bile are not correlated with their plasma levels	(Al-Kuraishy et al. 2025)
Bile acids act centrally by directly activating FXR and TGR5, which regulate neurotransmitter activity and related neurobehavioral responses. Bile acids also exert neuroprotective effects indirectly by inducing the expression of GLP-1 and FGF19	(Bazzari et al. 2019)
Bile acids have neuroprotective anti-inflammatory effects against AD development	(Collins 2023; D’Anniballe De Salles et al. 2026)

## Method and search strategy

This narrative review was developed through a structured literature search designed to capture key biochemical, experimental, and clinical evidence linking bile acid signaling to AD neuropathology. Relevant articles were identified by searching PubMed, Scopus, Embase, and Web of Science for publications from January 2000 to March 2025 using combinations of keywords and MeSH terms including “bile acids,” “Alzheimer’s disease,” “gut–brain axis,” “neuroinflammation,” “neurodegeneration,” “FXR,” “TGR5,” “GLP-1,” and “FGF19,” with Boolean operators applied to refine results. Additional studies were identified through manual screening of the reference lists of eligible reviews and original articles. Publications were included if they provided mechanistic, biochemical, or clinical insights into bile acid metabolism and receptor-mediated pathways relevant to AD across human studies, animal models, or in vitro systems. Non-peer-reviewed articles, conference abstracts, editorials, and case reports were excluded. Because the available evidence was heterogeneous in design and outcome measures, findings were synthesized narratively and organized into thematic sections addressing bile acid profile alterations in AD, receptor-dependent mechanisms, microbiota–bile acid interactions, and translational implications.

## Alterations of bile acid levels in AD

**Fig. 3:**
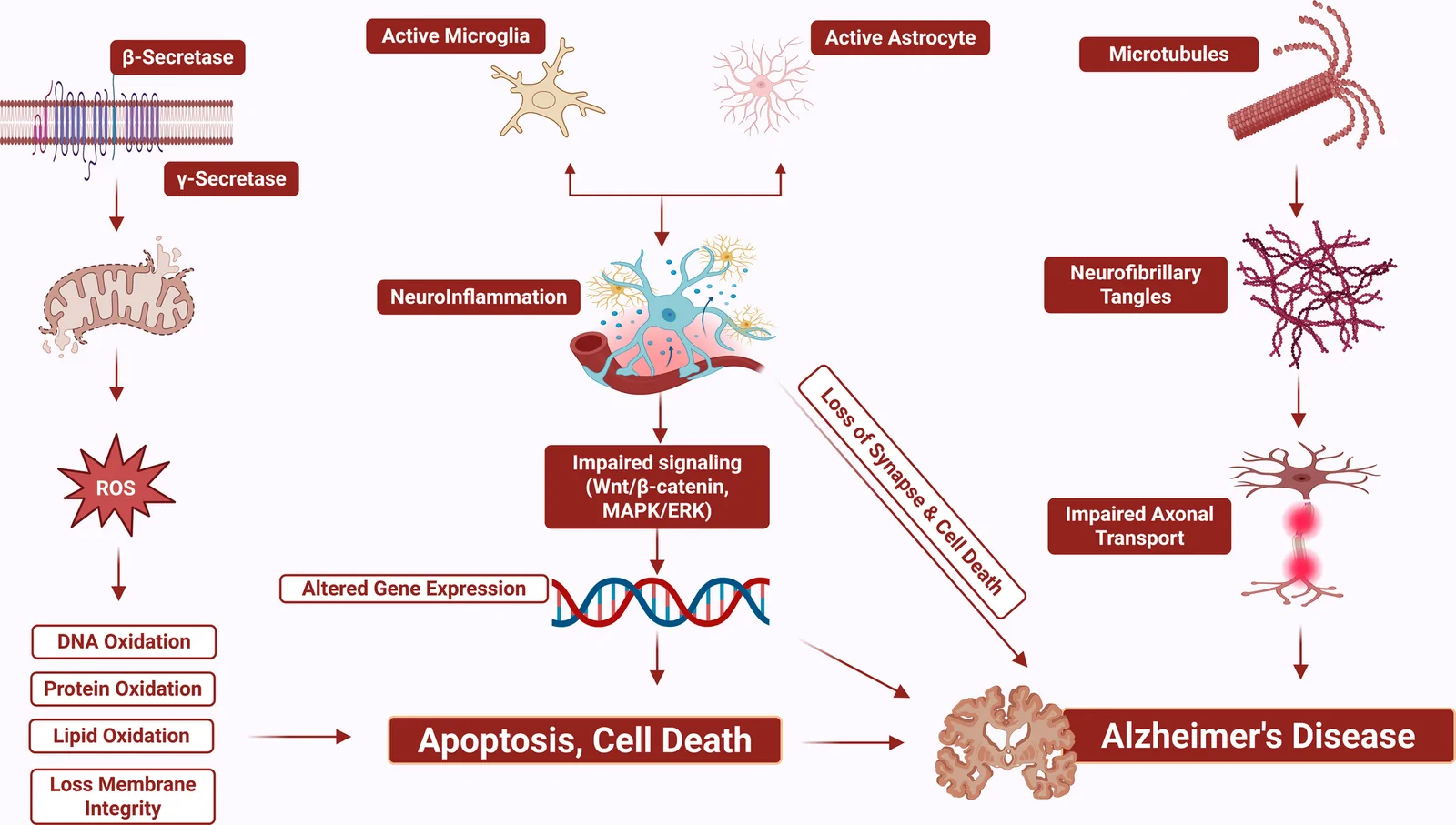
Core neuropathological mechanisms implicated in Alzheimer’s disease (AD). Amyloidogenic processing of the amyloid precursor protein (APP) by β- and γ-secretases promotes the generation of amyloid-β (Aβ), mitochondrial dysfunction, and excessive reactive oxygen species (ROS) production. ROS accumulation drives oxidative injury, including DNA, protein, and lipid oxidation, with disruption of membrane integrity and downstream transcriptional dysregulation. In parallel, activation of microglia and astrocytes amplifies neuroinflammation, impairing neuronal survival signaling pathways (including Wnt/β-catenin and MAPK/ERK cascades) and altering gene expression programs that promote apoptosis and neuronal cell death. Moreover, tau-associated cytoskeletal pathology is reflected by microtubule destabilization, formation of neurofibrillary tangles (NFTs), and impaired axonal transport, culminating in synaptic failure, progressive neuronal loss, and the clinical phenotype of Alzheimer’s disease

AD is the most common cause of dementia due to progressive brain neurodegeneration that is characterized by cognitive decline and memory impairment (DeMorrow 2019; Dey et al. 2025; Di Ciaula et al. 2022). Of note, aging, female sex, hypertension, ApoE4 carrier, hippocampal atrophy, exposure to neurotoxins, oxidative stress, obesity, and metabolic syndrome are the conceivable risk factors for the development of AD (Dionísio et al. 2015; Dolegowska et al. 2019; Dong et al. 2023). AD neuropathology is connected with the progressive accumulation of extracellular amyloid beta (Aβ) peptide and intracellular neurofibrillary tangles (NFTs) (Drucker 2022). Aβ is the primary source of reactive oxygen species (ROS) in AD, and Aβ-induced neurotoxicity is mediated by hydrogen peroxide and other ROS, which are inhibited by antioxidant administration (Du et al. 2022). Additionally, tau protein, which plays a critical role in regulating neuronal microtubules by preserving neuronal functions and axonal transport, is dysregulated in AD (Ehtezazi et al. 2023). Thus, the accumulation of Aβ and NFTs, together with oxidative stress and neuroinflammation, contributes to synaptic dysfunction and neuronal apoptosis through multiple interconnected mechanisms (Esan et al. 2022). Consequently, the pathogenesis of AD is complex and involves neuropathological factors. To contextualize the molecular targets affected by bile acids, see Fig.3.

In healthy adults, fasting serum total bile acid concentrations typically range from 2 to 10 µmol/L, although these values may vary with age and physiological conditions (Ferrell and Chiang 2021). In AD, alterations of bile acid profiles do not reflect a uniform increase in all bile acid species. Instead, metabolomics studies indicate a selective reduction in primary bile acids, accompanied by a rise in specific bacterially derived secondary bile acids, leading to an altered primary/secondary bile acid ratio (Ferrell and Chiang 2021). This imbalance has been strongly associated with cognitive decline, disrupted cholesterol metabolism, and gut–brain axis dysfunction in AD patients (Ferrell and Chiang 2021), suggesting gut–brain axis disruption and altered cholesterol metabolism, with potential implications for neuroinflammation (Ferrell et al. 2022). The temporal relationship between bile acid dysregulation and AD pathology remains incompletely defined. Available evidence suggests that bile acid alterations may function as parallel disease modifiers by influencing neuroinflammation, cholesterol metabolism, and gut–brain signaling (Ferrell et al. 2022). However, defects in bile acid–related gene expression observed in AD brains and shifts toward increased secondary bile acid production may also reflect downstream consequences of established neuropathology and gut–brain axis disruption (Al-Kuraishy et al. 2024). Thus, bile acid dysregulation likely represents a bidirectional and stage-dependent process rather than a purely initiating or purely secondary event (Ferrell and Chiang 2021). Consistently, serum levels of primary bile acids, including cholic acid (CA) and chenodeoxycholic acid (CDCA), as well as secondary bile acids such as deoxycholic acid (DCA) and lithocholic acid (LCA), and their glycine- and taurine-conjugated derivatives (e.g., GDCA, TDCA, GLCA, and TLCA), are altered in patients with early cognitive impairment, late cognitive impairment, and AD compared to healthy controls (Collins et al. 2023). Notably, increased secondary-to-primary bile acid ratios were associated with cognitive decline. Notably, PET imaging and metabolomic studies have demonstrated that bile acid dysregulation correlates with established AD biomarkers, including CSF Aβ42/40 ratio (amyloid pathology), p-tau217 (tau pathology), and neurofilament light chain (axonal degeneration), suggesting that alterations in bile acid metabolism may emerge during early biomarker-defined stages of the disease (Fiaschini et al. 2022; Fuchs and Trauner 2022). Therefore, bile acid metabolites in plasma, such as lithocholic acid, glycolithocholic acid, glycodeoxycholic acid, and glycochenodeoxycholic acid, could serve as diagnostic biomarkers and correlate with AD cognitive impairment (Collins et al. 2023; Ferrell and Chiang 2021).

In contrast, serum bile acid levels in AD patients did not differ significantly from those of healthy controls (Giridharan et al. 2022). Consequently, secondary bile acids are involved in the development of cognitive impairment and AD. Moreover, serum levels of hydrophobic secondary bile acids, particularly deoxycholic acid (DCA) and lithocholic acid (LCA), are elevated in patients with cognitive impairment and AD (Giridharan et al. 2022). Increased levels of these hydrophobic bile acids have been associated with BBB disruption and neuronal dysfunction in experimental models (Giridharan et al. 2022). Furthermore, alterations of cerebrospinal fluid (CSF) bile acid metabolites are associated with CSF tau and Aβ levels in AD patients compared with healthy controls (Fuchs and Trauner 2022).

**Fig. 4:**
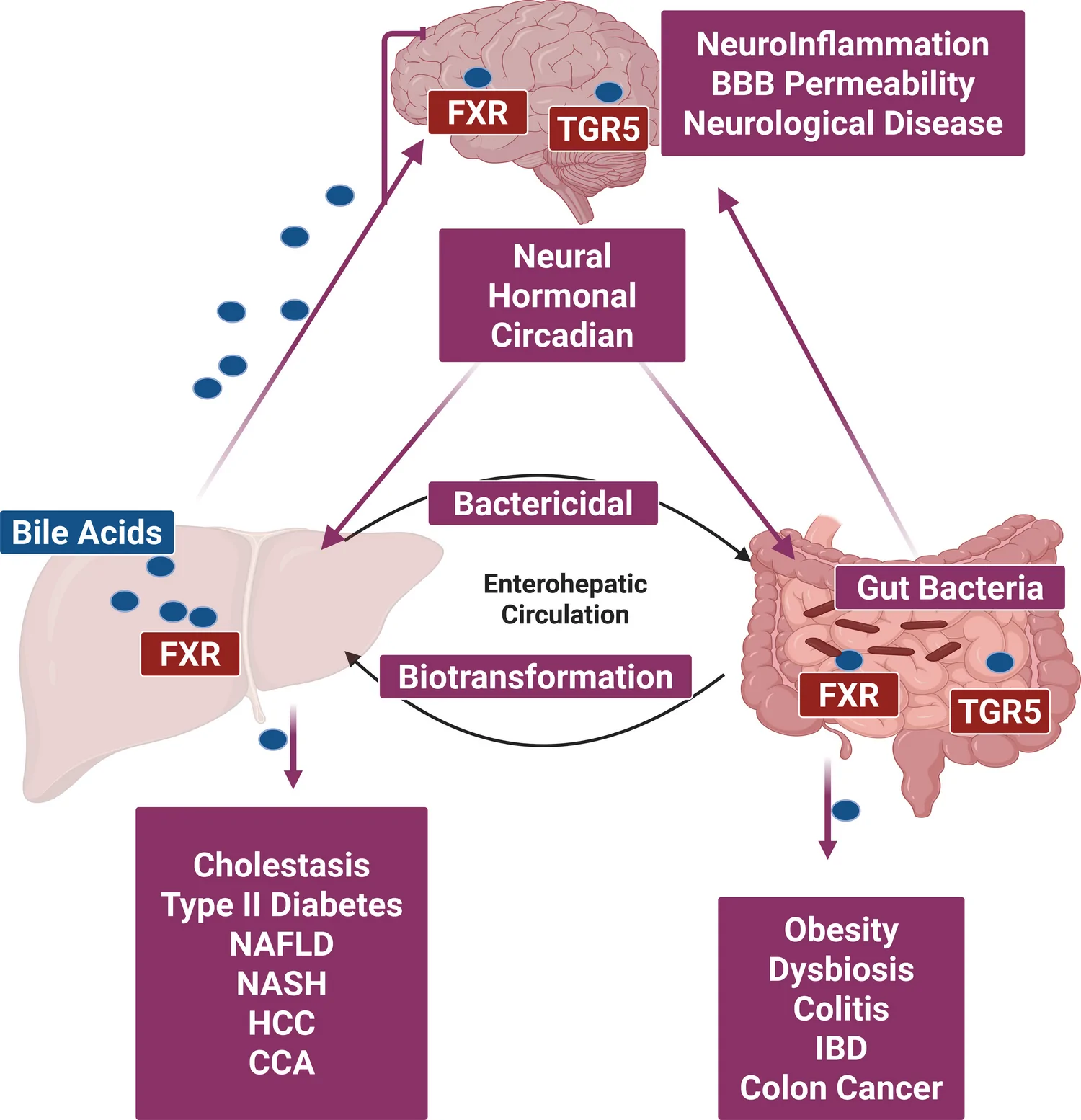
Bile acid–microbiota–brain signaling along the gut–liver–brain axis in health and disease. Primary and secondary bile acids (BAs) produced in the liver and modified by gut bacteria undergo enterohepatic circulation, shaping microbial ecology through bactericidal activity and, conversely, being diversified via microbial biotransformation. BAs function as endocrine-like signaling molecules that activate the nuclear receptor farnesoid X receptor (FXR) and the membrane G-protein-coupled receptor TGR5 in the liver, intestine, and central nervous system, thereby integrating metabolic and immune responses across organs. Peripheral and brain BA signaling influences neural, hormonal, and circadian regulation. At the same time, dysregulated receptor pathways contribute to neuroinflammation, altered blood–brain barrier (BBB) permeability, and increased susceptibility to neurological diseases. Disruption of BA homeostasis and gut microbial composition is implicated in hepatic and metabolic disorders (including cholestasis, type 2 diabetes, NAFLD/NASH, hepatocellular carcinoma [HCC], and cholangiocarcinoma [CCA]) as well as intestinal pathologies (including obesity, dysbiosis, colitis/IBD, and colorectal cancer), highlighting FXR/TGR5-mediated bile acid signaling as a unifying mechanistic link between gut and brain health

Interestingly, the role of bile acids in AD neuropathology has been recognized by numerous preclinical studies (D’Anniballe De Salles et al. 2026; Gofflot et al. 2007). It has been demonstrated that hydrophilic bile acids, such as tauroursodeoxycholic acid (TUDCA), play a neuroprotective role against Aβ-induced neurotoxicity in transgenic mouse models by inhibiting excessive microglial activation. TUDCA attenuates neurotoxicity by suppressing β- and γ-secretase activity, enhancing α-secretase-mediated nonamyloidogenic processing of APP, and preventing tau hyperphosphorylation (D’Anniballe De Salles et al. 2026; Gofflot et al. 2007). These mechanisms collectively contribute to reduced amyloid burden, neuroinflammation, and neuronal loss (D’Anniballe De Salles et al. 2026; Gofflot et al. 2007). Dependably, administration of TUDCA for 6 months restores cognitive impairment and memory deficit in the AD model (D’Anniballe De Salles et al. 2026). In addition, early treatment with TUDCA prevents the progression of AD by constraining the development of Aβ-induced neurotoxicity and neuroinflammation (D’Anniballe De Salles et al. 2026). On the other hand, lipophilic bile acids have detrimental effects on brain neurons by activating FXR, which inhibits brain bile acid synthesis, blocking NMDA receptors, interfering with the synthesis of brain 24S-hydroxycholesterol, and increasing brain cholesterol accumulation (Giridharan et al. 2022). Activation of FXR by bile acids prevents transactivation of brain FXRα, which increases cholesterol metabolism (Giridharan et al. 2022). Accumulation of brain cholesterol due to a defect in the synthesis of bile acids promotes the generation and aggregation of neurotoxic Aβ1–42 (Giridharan et al. 2022). Furthermore, overactivation of FXR in the hepatic encephalopathy model is associated with neurological deterioration (D’Anniballe De Salles et al. 2026; Giridharan et al. 2022). Therefore, bile acids may exert dual, context-dependent effects on AD progression, with hydrophilic bile acids conferring neuroprotection and hydrophobic secondary bile acids contributing to neurotoxicity and neuroinflammation (Fig.4, Table 2).

**Table 2:** Alterations of bile acids in AD

Studies	Findings	Ref
Clinical	A selective reduction in primary bile acids, accompanied by a rise in specific bacterially derived secondary bile acids, leads to an altered primary/secondary bile acid ratio	(Ferrell and Chiang 2021)
Clinical	Serum levels of primary bile acid (cholic acid) and secondary bile acids (deoxycholic acid), and their conjugated forms (glycine and taurine), are altered in patients with early cognitive impairment, late cognitive impairment, and AD compared with healthy controls	(Collins et al. 2023)
Clinical	PET imaging studies revealed that dysregulation of bile acids is correlated with biomarkers of neurodegeneration, such as p-tau217, the CSF Aβ42/40 ratio, and neurofilament light chain, in AD	(Fiaschini 2022; Fuchs and Trauner 2022)
Clinical	Serum levels of neurotoxic hydrophobic bile acids, which disrupt BBB integrity and induce neuronal dysfunction by reducing brain oxysterol synthesis and increasing Aβ production, are elevated in patients with cognitive impairment and AD	(Giridharan et al. 2022)
Clinical	Alterations of CSF bile acid metabolites are associated with CSF tau and Aβ levels in AD patients compared with healthy controls	(Fuchs and Trauner 2022)
Preclinical	The hydrophilic bile acids (TUDCA) play a neuroprotective role against Aβ-induced neurotoxicity in transgenic mouse models by inhibiting excessive microglial activation	(D’Anniballe De Salles et al. 2026; Gofflot et al. 2007)
Preclinical	TUDCA restores cognitive impairment and memory deficit in the AD model by inhibiting Aβ-induced neurotoxicity and neuroinflammation	(D’Anniballe De Salles et al. 2026)
Preclinical	The lipophilic bile acids have detrimental effects on brain neurons by activating FXR, which blocks NMDA receptors, interferes with the synthesis of brain 24S-hydroxycholesterol, and increases the accumulation of brain cholesterol. Accumulation of brain cholesterol due to a defect in bile acid synthesis promotes the generation and aggregation of neurotoxic Aβ1–42	(Giridharan et al. 2022)

## Direct effects of bile acids on AD neuropathology

Of note, a small fraction of bile acids is synthesized locally within the brain (Gu et al. 2023). Within the brain, bile acids, via activation of LXRs and TGR5, regulate brain cholesterol homeostasis and synaptic plasticity (Bazzari et al. 2019). It has been demonstrated that primary bile acids exhibit anti-inflammatory and neuroprotective effects in various brain disorders (Honarpisheh et al. 2020). Nevertheless, secondary bile acids have neurotoxic effects by inducing BBB injury and neuroinflammation, including hepatic encephalopathy (Honarpisheh et al. 2020). Therefore, the direct impact of bile acids on the brain is contradictory and could be receptor-dependent (Table 3).

**Table 3:** Direct effects of bile acids on AD neuropathology

Studies	Findings	Ref
Preclinical	Primary bile acids may indirectly influence LXR-regulated neuroprotective pathways by modulating cholesterol metabolism and receptor crosstalk	(Ferrell et al. 2022)
Preclinical	Modulation of bile acid signaling has been associated with improved brain insulin sensitivity through mechanisms linked to LXR-regulated metabolic pathways	(Gofflot et al. 2007)
Preclinical	Pharmacological activation of LXRs has been shown to alleviate AD-related neuropathology by enhancing Aβ clearance, reducing neuroinflammation, and improving cholesterol homeostasis	(Li et al. 2024)
Preclinical	Dysregulated bile acid profiles, particularly elevated hydrophobic secondary bile acids, have been associated with neuroinflammatory signaling in experimental models	(Li et al. 2024)
Preclinical	Activation of TGR5 by bile acids regulates hippocampal glutamate release by prompting the expression and signaling pathway of cAMP-PKA-CREB	(Maselli and Camilleri 2021)
Preclinical	Gut microbiota–mediated bile acids, mostly ursodeoxycholic acid, enhance cognitive function and neurological function through the activation of TGR5 and inhibition of the NLRP3 inflammasome in ischemic stroke models	(Monteiro-Cardoso et al. 2021)
Preclinical	Natural bear bile powder, via activation of TGR5, inhibits the release of proinflammatory cytokines from activated microglia in BV2 microglia	(Mosińska et al. 2018)
Preclinical	Chenodeoxycholic acid attenuates AlCl_3_-induced AD in rats via TGR5 activation	(Mu et al. 2022)
Preclinical	An increase in deoxycholic acid in the brains of mice with early AD induces upregulation of brain TGR5 and the development of cognitive impairment by enhancing APP processing and γ-secretase activation	(Mulak 2021)
Preclinical	Increased plasma levels of secondary bile acids, accompanied by reduced primary bile acids, contribute to AD development via maladaptive TGR5 signaling	(Belka et al. 2024)

### Role of LXR in AD

LXRs are the central nuclear receptors expressed peripherally and centrally, regulating inflammation and cholesterol metabolism (Huang et al. 2022). Two isoforms of LXRs, including LXRα and LXRβ, form heterodimers with PPARs (Huang et al. 2022). Both LXRα and LXRβ are expressed in the brain; however, LXRβ is highly expressed, approximately 3–5 times more than LXRα (Kim et al., 2021), suggesting that LXRβ mainly mediates bile acid action in the brain (Kaczmarczyk et al. 2017). LXRα is primarily expressed in tissues with high metabolic activity, including the intestine, adipose tissue, liver, and kidney (Kakiyama et al. 2013). In particular, LXRα and LXRβ share target genes, allowing them to compensate for each other in regulating the cholesterol transporters ABCA1 and ABCG5/8. LXRα and LXRβ share 77% homology in their ligand-binding and DNA-binding domains in mice (Kakiyama et al. 2013). LXRs, along with PPARs, are the primary nuclear type II receptors involved in regulating brain cholesterol (Kakiyama 2013).

It has been demonstrated that LXRs exhibit neuroprotective effects against the development and progression of AD by reducing the production and accumulation of Aβ (Kaur et al. 2021). LXRs play a crucial role in AD pathogenesis by regulating APP processing and reducing Aβ neurotoxicity in mice (Kaur et al. 2021). Consistently, LXR agonists inhibit microglial activation and the release of proinflammatory cytokines, thereby inhibiting the progression of neuroinflammation in AD (Kaur et al. 2021; Kawamata et al. 2003). Furthermore, LXR agonists, such as T0901317 and GW3965, decreased the levels and insoluble Aβ accumulation. In particular, T0901317 reduces Aβ_42_levels in the hippocampus and reverses cognitive impairment in transgenic mice (Kim et al. 2021). In addition, LXR agonists decrease Aβ levels in the interstitial fluid of transgenic mice by increasing the clearance of brain-insoluble Aβ. Moreover, the LXR agonist T0901317 enhances microglial phagocytosis of Aβ but not pericyte phagocytosis in transgenic mice (Kim et al. 2021). Notably, T0901317 enhances neurogenesis in cholinergic neurons, thereby mitigating cognitive impairment and memory loss in AD (Kim et al. 2021). Recently, Martens et al. demonstrated that the LXR agonist 22-ketositosterol attenuates the progression of AD neuropathology by increasing cholesterol efflux in cultured primary neurons and in transgenic APP mice.

Additionally, 22-ketosterol inhibits microglia and astrocytes in the hippocampus and cerebral cortex, without affecting Aβ accumulation, in transgenic APP mice (Kim et al. 2021), suggesting that LXR’s anti-inflammatory effects are independent of Aβ production and accumulation. Furthermore, activation of brain LXRs reduces brain cholesterol and caveolin-1 levels by activating the ABCA1 transport system, ultimately leading to reduced Aβ production and accumulation (Kawamata et al. 2003; Larabi et al. 2023). Interestingly, abnormal cholesterol metabolism is associated with the development and progression of AD neuropathology (Larabi et al. 2023). Caveolin-1 is a scaffolding protein that regulates mitochondrial function, though abnormal expression of caveolin-1 is linked with the pathogenesis of AD. Therefore, LXRs, by modulating brain cholesterol and caveolin-1, improve synaptic plasticity and cognitive function (Lee et al. 2023).

Furthermore, preclinical studies have shown that pharmacological LXR agonists improve cognitive impairment in AD models by enhancing Aβ clearance and reducing senile plaque burden (Gofflot et al. 2007). Given the close metabolic relationship between bile acid synthesis and cholesterol homeostasis, primary bile acids may influence LXR-regulated pathways indirectly through receptor crosstalk and modulation of cholesterol metabolism. However, direct activation of LXR by CA or CDCA within the CNS has not been conclusively demonstrated (Ferrell et al. 2022).

**Fig. 5:**
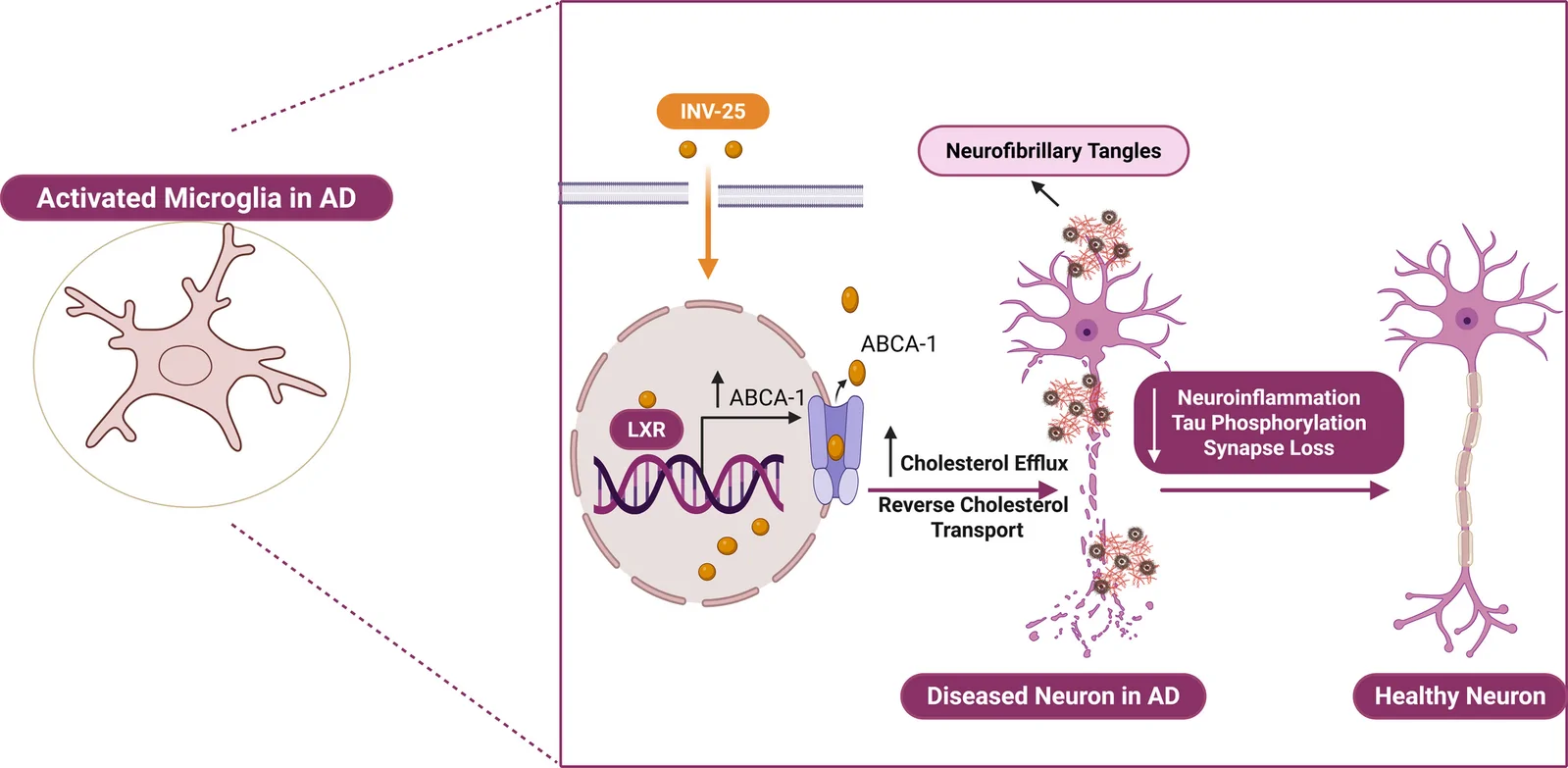
LXR–ABCA1-mediated cholesterol efflux as a microglia-targeted neuroprotective strategy in Alzheimer’s disease (AD). In AD, activated microglia exhibit dysregulated lipid handling and sustain a proinflammatory milieu, contributing to synaptic dysfunction and neurodegeneration. Pharmacological activation of liver X receptor (LXR) by the synthetic agonist INV-25 promotes transcriptional upregulation of the cholesterol transporter ATP-binding cassette transporter A1 (ABCA1). Increased ABCA1 expression enhances cellular cholesterol efflux and supports reverse cholesterol transport, improving lipid homeostasis within the neuroimmune environment. Restoration of cholesterol trafficking is associated with attenuation of AD-related pathology, including reduced neuroinflammation, decreased tau phosphorylation and neurofibrillary tangle burden, and mitigation of synapse loss, ultimately preserving neuronal integrity and shifting the balance from a diseased neuron phenotype toward a healthier neuronal state

Conversely, dysregulated bile acid profiles, particularly elevated levels of hydrophobic secondary bile acids, have been associated with neuroinflammatory signaling in conditions such as experimental liver injury. However, these effects are not necessarily mediated through direct LXR activation. Activation of LXRs by established agonists, particularly within the hippocampus and prefrontal cortex, has been shown to enhance neurotransmission, neurogenesis, synaptic plasticity, and neuroendocrine regulation (Gofflot 2007). Consequently, changes in bile acids, accompanied by a significant reduction in the primary bile acids, attenuate the neuroprotective signaling mediated by LXRs. Moreover, LXRs enhance brain insulin sensitivity by increasing the expression of miR-7 and the RNA-binding protein, which in turn modulate the expression of insulin receptors, insulin receptor substrate 1, and insulin-degrading enzyme (IDE) (Li et al. 2021). Brain insulin resistance contributes to AD progression by increasing*APP*gene expression and, in turn, overproducing insoluble Aβ (Li et al. 2021). Brain insulin resistance also triggers a deficiency in neuronal metabolism, leading to oxidative stress and associated neurodegeneration (Li et al. 2021). It has been reported that bile acids, by activating brain LXRs, mitigate brain insulin resistance and the progression of AD (Li et al. 2024). Interestingly, FGF19/FGFR signaling suppresses bile acid production in the liver through a canonical negative feedback mechanism mediated by intestinal FXR activation. Upon bile acid–induced FXR activation in enterocytes, FGF19 (FGF15 in rodents) is released and signals via hepatic FGFR4 to inhibit CYP7A1 expression, thereby reducing bile acid synthesis (Li et al. 2024). While TGR5 and LXR participate in broader bile acid–related metabolic and inflammatory signaling pathways, they are not the primary mediators of this feedback inhibition (Fig.5).

### Role of TGR5 in AD

TGR5 is a G-protein-coupled receptor superfamily member widely expressed across tissues, including the brain. TGR5 was initially discovered in 2003 as an inhibitor of lipopolysaccharide (LPS)-induced macrophage activation (Liu et al. 2018). TGR5 is the first recognized bile acid receptor, expressed widely in many brain areas, particularly the frontal cortex and hippocampus (Lund et al. 2020). TGR5 is regarded as a metabolic regulator of bile acid and energy homeostasis, as well as glucose metabolism, by modulating ERK and NF-κB (Bazzari et al. 2019). In the CNS, TGR5 expression is low, though it is comparatively high in the hippocampus, hypothalamus, pituitary, and cerebral cortex (Bazzari et al. 2019). TGR5 is activated by bile acids and other endogenous agonists, such as neurosteroids, as well as by exogenous agonists, including ciprofloxacin antibiotics and natural terpenoids (Bazzari et al. 2019). TGR5 signaling is mediated by activating cAMP, which inhibits NF-κB expression (Liu et al. 2018). Furthermore, TGR5 regulates the excitability of GABAergic neurons in the brain via ERK signaling (Luo et al. 2022). Activated TGR5 mediates the satiety effect of bile acids in the hypothalamus. Consistently, direct administration of bile acids or TGR5 agonists into the CNS induces anorexia in mice (MahmoudianDehkordi et al. 2019). In addition, under physiological conditions, TGR5 regulates mood and cognitive functions (Luo et al. 2022). However, dysregulation of brain TGR5 is linked with the development of depressive symptoms in mice (Luo et al. 2022). Significantly, the expression of brain TGR5 is highly reduced in chronic stress (Luo et al. 2022). Additionally, TGR5 reduces oxidative stress and neuronal apoptosis by suppressing caspase-3 expression (Martens et al. 2025). Findings from a preclinical study revealed that the TGR5 agonist INT-777 attenuates streptozotocin (STZ)-induced cognitive impairment in mice by promoting neurogenesis, inhibiting neuronal apoptosis, and modulating pyramidal neuron firing (Lund et al. 2020). Meaningfully, activation of TGR5 by bile acids regulates hippocampal glutamate release by prompting the expression and signaling pathway of cAMP-PKA-CREP (Maselli and Camilleri 2021). Furthermore, activated brain TGR5 alleviates hepatic encephalopathy-induced neuroinflammation by inhibiting microglial activation and reducing the release of proinflammatory cytokines (Mayer et al. 2022). Similarly, the TGR5 agonist INT-777 reduces neuroinflammation and neurodegeneration by inhibiting microglia in the MPTP PD model (Mertens et al. 2017).

Additionally, INT-777 reduces Aβ-induced neurotoxicity by inhibiting the release of proinflammatory cytokines, caspase activation, and regulating neuronal apoptosis in the hippocampus and frontal cortex. Zhang et al. (Monteiro-Cardoso et al. 2021) demonstrated that gut microbiota–mediated bile acids, mostly ursodeoxycholic acid, enhance cognitive function and neurological function through the activation of TGR5 and inhibition of the NLRP3 inflammasome in a mouse ischemic stroke model. These findings identified the neuroprotective effects of TGR5 in preventing the development and progression of AD by inhibiting neuroinflammation. Regularly, diverse studies have shown that activation of brain TGR5 inhibits signaling pathways, such as NF-κB, which is implicated in the pathogenesis of AD. For example, natural bear bile powder, via activation of TGR5, inhibits the release of proinflammatory cytokines from activated microglia in BV2 microglia (Mosińska et al. 2018). Moreover, tomato juice has been shown to improve cognitive impairment by inhibiting neuroinflammation via TGR5 activation (Mosińska et al. 2018). Furthermore, chenodeoxycholic acid attenuates AlCl_3_-induced AD in rats via different mechanisms, including TGR5 activation (Mu et al. 2022).

**Fig. 6:**
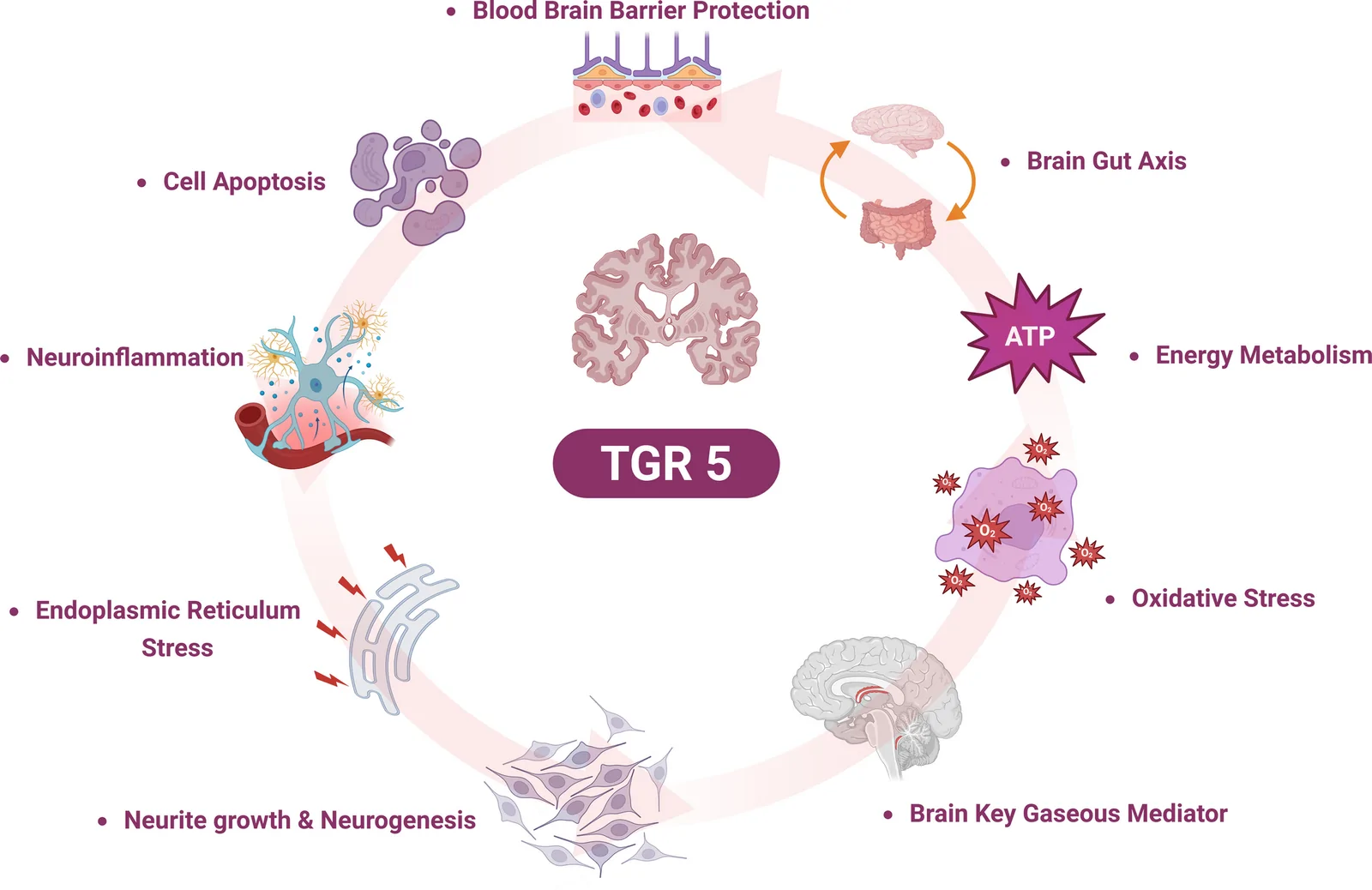
Neuroprotective and metabolic functions of TGR5 signaling in the central nervous system. Activation of the bile acid–sensing G-protein-coupled receptor TGR5 (GPBAR1) engages a broad spectrum of cellular programs relevant to brain homeostasis and neurodegeneration. TGR5 signaling contributes to blood–brain barrier (BBB) protection, modulation of the brain–gut axis, and regulation of energy metabolism through enhanced mitochondrial activity and ATP production. In parallel, TGR5 activation mitigates oxidative stress, attenuates endoplasmic reticulum (ER) stress, and suppresses neuroinflammatory responses, thereby limiting proapoptotic cascades and cell death. Beyond its anti-inflammatory and cytoprotective roles, TGR5 signaling has been linked to neurite outgrowth and neurogenesis. It may influence central neuromodulatory pathways, including the regulation of key gaseous mediators within the brain. Collectively, these pleiotropic actions position TGR5 as an integrative molecular node connecting bile acid signaling to neurovascular integrity, metabolic control, and neuronal resilience

However, an increase in deoxycholic acid in the brains of mice with early AD induces upregulation of brain TGR5 and the development of cognitive impairment. The activated TGR5 enhances APP processing and γ-secretase activation (Mulak 2021). Findings from an experimental study showed that hepatic cholesterol synthesis and primary bile acid production are reduced. In contrast, the production of secondary bile acids is increased in the mouse AD model (Belka et al. 2024), suggesting that these bile acids exert detrimental effects via TGR5 activation. Increased plasma levels of secondary bile acids, accompanied by reduced primary bile acids, contribute to AD development via maladaptive TGR5 signaling. Accordingly, bile acids produce differential downstream effects following TGR5 activation (Belka et al. 2024) (Fig.6).

**Fig. 7:**
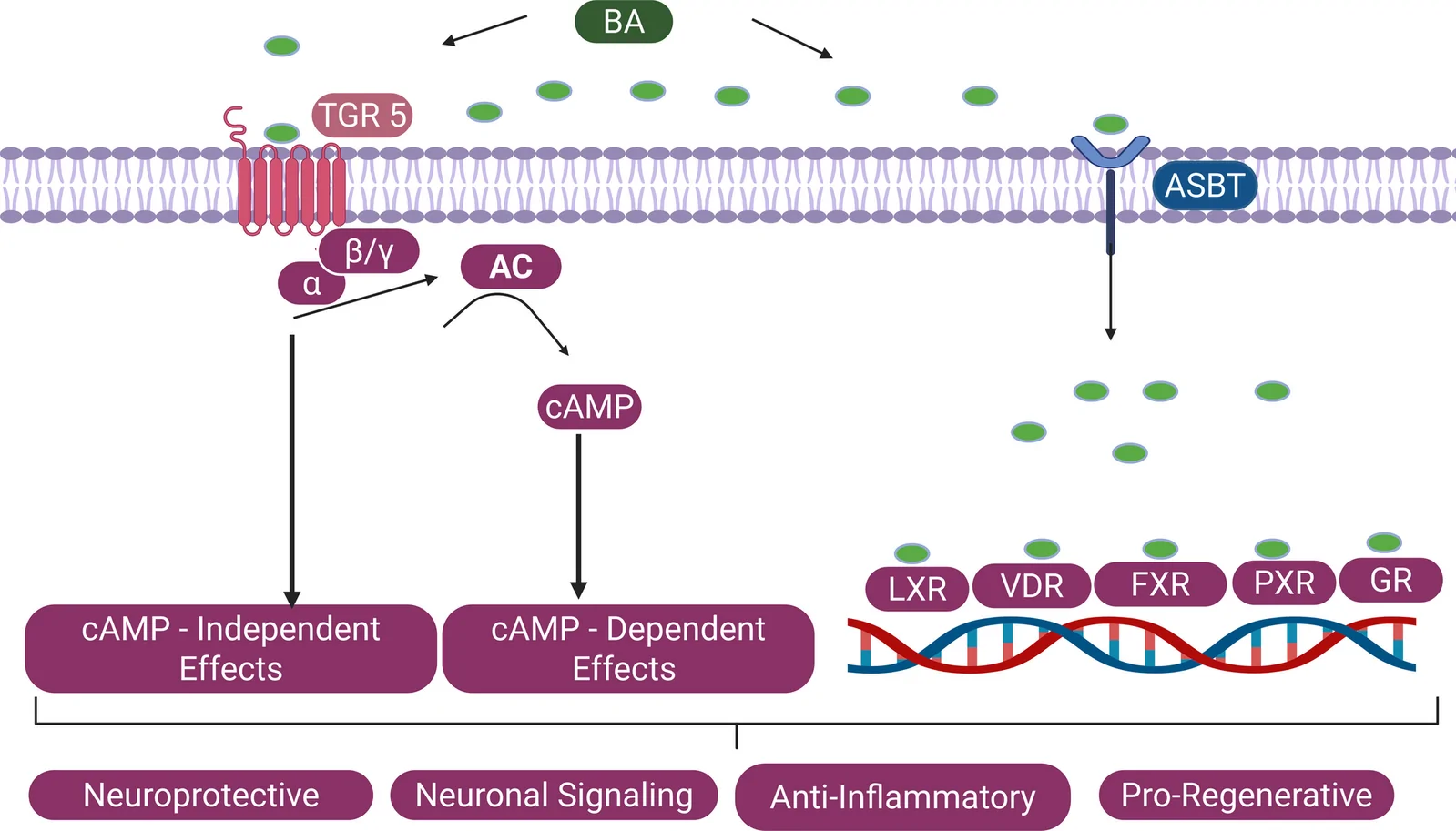
Bile acid receptor–dependent signaling pathways mediating neuroimmune and neuroprotective effects. Bile acids (BAs) act as pleiotropic signaling molecules that activate membrane and nuclear receptors to regulate inflammatory and neuronal homeostasis. At the plasma membrane, BAs activate the G-protein-coupled receptor TGR5 (GPBAR1), stimulating Gα/βγ signaling to engage adenylyl cyclase (AC) and elevate intracellular cAMP, thereby initiating cAMP-dependent cascades as well as cAMP-independent downstream effects. In parallel, BA uptake into cells is facilitated by the apical sodium-dependent bile acid transporter (ASBT), enabling intracellular access of BAs to ligand-activated transcriptional regulators, including FXR, GR, LXR, PXR, and VDR. Together, these coordinated signaling mechanisms converge to promote anti-inflammatory actions, modulate neuronal signaling, and support neuroprotective and proregenerative responses, highlighting bile acid receptor networks as key modulators of CNS resilience

Bile acids depict the major intracellular signaling pathways activated by TGR5, highlighting its role in modulating cAMP-dependent signaling, oxidative stress, inflammatory responses, and neuronal survival within the central nervous system (Belka et al. 2024). Primary bile acids activate TGR5, FXR, and related receptors, leading to subsequent neuroprotective outcomes (Belka et al. 2024). In contrast, specific hydrophobic secondary bile acids, such as deoxycholic acid and lithocholic acid, have been associated with detrimental consequences, including neuroinflammation and BBB disruption, partly through maladaptive TGR5 signaling. Importantly, ursodeoxycholic acid (UDCA) and TUDCA exert well-established neuroprotective, anti-inflammatory, and antiapoptotic effects (Mertens et al. 2017). Thus, TGR5 mediates ligand-specific and context-dependent impact, rather than acting as an intrinsically neuroprotective or neurotoxic receptor (Fig.7).

## Indirect effects of bile acids on AD neuropathology

### Gut–brain axis

The gut–brain neuronal axis is a bidirectional communication network that integrates microbial metabolites, enteroendocrine signaling, immune mediators, and direct neuronal pathways (Nasb et al. 2024). In addition to systemic metabolic and inflammatory signals, gut-derived factors, including bile acids, modulate vagal afferent transmission and enteric nervous system activity, thereby influencing central synaptic function, neuroinflammation, and cognitive processes. Dysregulation of this integrated axis has been implicated in AD and other neurodegenerative disorders (Nasb et al. 2024). Additionally, brain diseases such as AD alter the composition of the gut microbiota (Nguyen et al. 2020). It has been revealed that alterations in gut microbiota and the development of gut dysbiosis impair intestinal barrier integrity, leading to the subsequent transport of gut metabolites across the BBB into the brain. Bile acids promote the maturation and diversity of the gut microbiota through their antimicrobial and detergent effects (Nho et al. 2019). Bile acids also maintain intestinal barrier integrity through the FXR-dependent pathway (Perino et al. 2021). However, bile acids via TGR5 signaling preserve the gut–brain axis by inducing the expression of the neuroprotective GLP-1 and FGF19 (Ren et al. 2022).

It has been shown that gut dysbiosis via the gut–brain axis triggers AD neuropathology by inducing the production and accumulation of neurotoxic Aβ (Nho et al. 2019). Importantly, gut dysbiosis triggers Aβ production and accumulation in the myenteric plexus before Aβ accumulation in the brain and associated neuroinflammation in mice (Riggs et al. 2024). Notably, Aβ deposits were detected in the intestinal autopsies of AD patients and transgenic mice (Riggs et al. 2024). Therefore, intestinal Aβ pathology precedes brain Aβ pathology in AD (Romero-Ramírez and Mey 2024).

Furthermore, gut dysbiosis is associated with alterations in gut metabolites, characterized by reduced levels of the neuroprotective short-chain fatty acids (SCFAs) and increased levels of the neurotoxic trimethylamine N-oxide (TMAO) (Romero-Ramírez and Mey 2024). Specific gut bacterial taxa are responsible for the 7α-dehydroxylation of primary bile acids into secondary bile acids. Members of*Clostridium* cluster XIVa, particularly *Clostridium scindens*, *Clostridium hylemonae*, and *Clostridium hiranonis*, play a central role in converting cholic acid (CA) and chenodeoxycholic acid (CDCA) into deoxycholic acid (DCA) and lithocholic acid (LCA), respectively. These species express the bile acid–inducible operon, which encodes the enzymatic machinery required for secondary bile acid production. Dysregulation of these taxa may therefore shift bile acid composition toward more hydrophobic and potentially neuroactive species (Sandoval-Hernández et al. 2015).

These bile acids, particularly deoxycholic acid (DCA) and lithocholic acid (LCA), are known to induce the release of proinflammatory cytokines, intestinal inflammation, and barrier dysfunction (Romero-Ramírez and Mey 2024). Case–control studies have shown that microbial dysbiosis alters lipid metabolism, including upregulation of bacterial species responsible for the synthesis of hydrophobic secondary bile acids. For example, bile acid analysis in the appendix of patients with PD revealed increased levels of DCA and LCA compared to healthy controls (Shibuya et al. 2024). Further proteomic and transcriptomic analyses of the appendix and ileum corroborated these findings, highlighting disrupted bile acid regulation and cholesterol homeostasis. Notably, some bile acids detected in brain tissue cannot be explained by local synthesis, suggesting a gut microbiota origin and transport to the brain. Although much of this evidence derives from PD, similar gut microbiota–bile acid mechanisms are increasingly implicated in AD and may contribute to cognitive decline (Shibuya et al. 2024).

Furthermore, secondary bile acids and toxic metabolites resulting from gut dysbiosis can affect the neural activity of the vagus nerve. The vagus nerve is a significant part of the parasympathetic nervous system; it contains 80% efferent fibers and 20% afferent fibers (Siddeeque et al. 2024; Singh and Aran et al. 2026). The vagus nerve can detect various metabolites produced by the gut microbiota and transmit them to the brain. It has a potent anti-inflammatory effect against intestinal inflammation and neuroinflammation. Therefore, the vagus nerve is an interface between the gut and the brain (Siddeeque et al. 2024; Singh and Aran 2026). Of note, the vagus nerve prevents microglia activation and the development of neuroinflammation in AD. Additionally, the vagus nerve has a cognitive-enhancing effect by inducing synaptic plasticity, thereby attenuating the development and progression of AD (Singh and Aran 2026). However, vagus nerve neurotransmission in AD is reduced due to peripheral and central Aβ (Siddeeque et al. 2024; Singh and Aran 2026).

Hydrophilic bile acids such as ursodeoxycholic acid (UDCA) and tauroursodeoxycholic acid (TUDCA) have demonstrated neuroprotective, anti-inflammatory, and antiapoptotic effects in preclinical AD models, including attenuation of Aβ pathology and preservation of cognitive function. However, despite encouraging experimental data, clinical evaluation of UDCA/TUDCA in AD remains limited, and further controlled trials are required to determine efficacy and optimal dosing strategies (Sodhi and Singh 2013).

Therefore, dysregulation of the gut–brain axis, driven by gut dysbiosis and associated bile acid alterations, may contribute to the development and progression of AD. Accordingly, therapeutic strategies aimed at restoring primary bile acid signaling while limiting excessive secondary bile acid production may represent a promising avenue for modulating gut–brain axis dysfunction in AD (Sodhi and Singh 2013). Thus, restoration of gut microbial balance through probiotics, prebiotics, and selected dietary supplements has emerged as a potential therapeutic strategy in AD. Probiotic and synbiotic interventions have been reported to modulate microbial composition, enhance short-chain fatty acid production, influence bile acid metabolism, and improve intestinal barrier integrity. These effects may reduce systemic and central neuroinflammation and support cognitive function via the gut–brain axis (Sodhi and Singh 2013). Although current clinical evidence remains preliminary and heterogeneous, microbiota-targeted interventions represent a promising adjunctive approach in the management of AD (Sodhi and Singh 2013).

### GLP-1

GLP-1 is a gut hormone released from L cells involved in glucose-stimulated insulin secretion and inhibition of glucagon release (Song et al. 2024). GLP-1 has multiple physiological and pharmacological properties (Song et al. 2024). GLP-1 improves blood glucose homeostasis and reduces appetite, thereby effectively preventing diabetes and obesity (Song et al. 2024). Furthermore, GLP-1 has neuroprotective effects against AD and other neurodegenerative diseases by inhibiting neuroinflammation and oxidative stress (Tang et al. 2023). GLP-1, via activation of the GLP-1 receptor (GLP-1R), which is also widely expressed in the CNS, improves cognitive function (Song et al. 2024). It has been shown that GLP-1R agonists reduce AD neuropathology by decreasing the accumulation of Aβ and tau proteins and by enhancing the expression of the neuroprotective brain-derived neurotrophic factor (BDNF) in AD models (Tian et al. 2023). Therefore, augmentation of brain GLP-1 signaling may mitigate AD pathogenesis.

Furthermore, bile acids trigger GLP-1 release from intestinal L cells by activating TGR5 and FXR and by increasing ASBT expression in mice. TUDCA and taurolithocholate increase GLP-1 secretion via cAMP signaling in primary intestinal cultures (Vargas-Caballero et al. 2022). Therefore, bile acids have been suggested to be effective in managing diabetes by activating GLP-1 secretion through the TGR5/LXR signaling pathway. A case–control study showed that postprandial bile acid levels are positively correlated with GLP-1 and gut hormone peptide YY and negatively correlated with ghrelin. Many studies have confirmed the effects of TGR5 and LXR on GLP-1 release from L cells (Varma et al. 2021; Wang et al. 2021). For example, the natural product curcumin, a TGR5 agonist and FXR antagonist, has been shown to enhance GLP-1 release from L cells in male mice (Varma et al. 2021). Consistently, suppression of intestinal LXR with thyroid hormone improves GLP-1 release from L cells in mice (Wang et al. 2021). In parallel, GLP-1 receptor agonists—currently approved for the treatment of type 2 diabetes and obesity—have shown neuroprotective effects in AD models and early clinical investigations. Given their established safety profile and capacity to modulate central insulin signaling and neuroinflammation, GLP-1-based therapies represent a promising translational avenue targeting bile acid–mediated gut–brain signaling pathways. Future studies should determine whether modulation of bile acid–GLP-1 signaling confers disease-modifying effects in biomarker-defined stages of AD.

### FGF19

FGF19 is a gut-derived circulating growth factor that regulates lipid and glucose metabolism, as well as the homeostasis of vitamin D3 (Wang et al. 2023). The FGF19 protein is a subclass of FGF proteins, including FGF19, FGF21, and FGF23 (Wang et al. 2023). The FGF19 has a lower affinity to the FGF receptor than other growth factors (Wu et al. 2024). FGF19 signaling regulates memory and cognitive function, and reduced expression of brain FGF19 mRNA is linked to the development of depression and cognitive impairment (Xian et al. 2018). FGF19 exerts a neuroprotective effect by reducing Aβ production and accumulation, as well as associated neurodegeneration (Xing et al. 2023). It has been demonstrated that FGF19/FGFR signaling enhances hippocampal neurogenesis by stimulating BDNF, thereby mitigating vascular dementia and cognitive impairment in animal models. Notably, FGF19/FGFR signaling is highly deregulated in AD and other types of dementia (Yan et al. 2022). Therefore, activating the brain FGF19/FGFR signaling can attenuate AD progression. It has been demonstrated that FGF19/FGFR signaling suppresses bile acid production in the liver through a negative feedback mechanism involving the TGR5/LXR signaling pathway. Inhibition of FGF19/FGFR signaling results in overproduction of bile acids (Ferrell et al. 2022). However, bile acids stimulate FGF19 release from the intestine, which activates hypothalamic FGFR in mice via the gut–brain axis. Hypothalamic FGFR enhances the neuroprotective release of NPY, which regulates brain insulin sensitivity (Ye et al. 2024). Thus, bile acids activate peripheral FGF19/FGFR signaling and, via the gut–brain axis, attenuate cognitive decline in AD. Furthermore, FGF19/FGFR signaling suppresses bile acid production in the liver through a negative feedback mechanism. The negative feedback regulation of bile acid synthesis is primarily mediated through the FGF19/15-FXR pathway (Zhai et al. 2023).

### Serotonin

Serotonin or 5-hydroxytryptamine (5-HT) is a neurotransmitter expressed in the CNS and peripheral nervous system, as well as the gut and platelets (Zhang et al. 2023). About 90–95% of the total body 5-HT is produced by the enterochromaffin cells of the intestinal mucosa. 5-HT release from enterochromaffin cells is activated by acetylcholine and inflammation (Zhang et al. 2023, 2024). 5-HT is critical in preventing neurodegeneration, oxidative stress, and neuroinflammation in AD and other neurodegenerative diseases (Zhang et al. 2024). Interestingly, 5-HT expression is highly reduced in the brains of AD in both animals and humans, which is linked with cognitive impairment and progression of AD neuropathology (Zheng et al. 2025). Furthermore, 5-HT stimulates gallbladder contraction and bile acid excretion and induces ASBT expression in the intestines. Additionally, 5-HT mediates the trophic effect of TGR5 on L cells, leading to the release of GLP-1 (Zhou et al. 2021). Inhibition of 5-HT signaling attenuates bile acid release by modulating FXR (Zhou et al. 2023). In addition, TGR5 activates the release of 5-HT from L cells. Findings from a preclinical study demonstrated that*TGR5*gene knockout reduced 5-HT release from L cells (Zuo et al. 2019). Conversely, FXR inhibits the release of 5-HT both peripherally and centrally. In FXR knockout mice, the depressive symptoms are low due to an increase in brain 5-HT neurotransmission. Therefore, bile acids, by modulating TGR5/FXR, increase gut 5-HT, which in turn attenuates the development and progression of AD via the gut-brain axis.

**Fig. 8:**
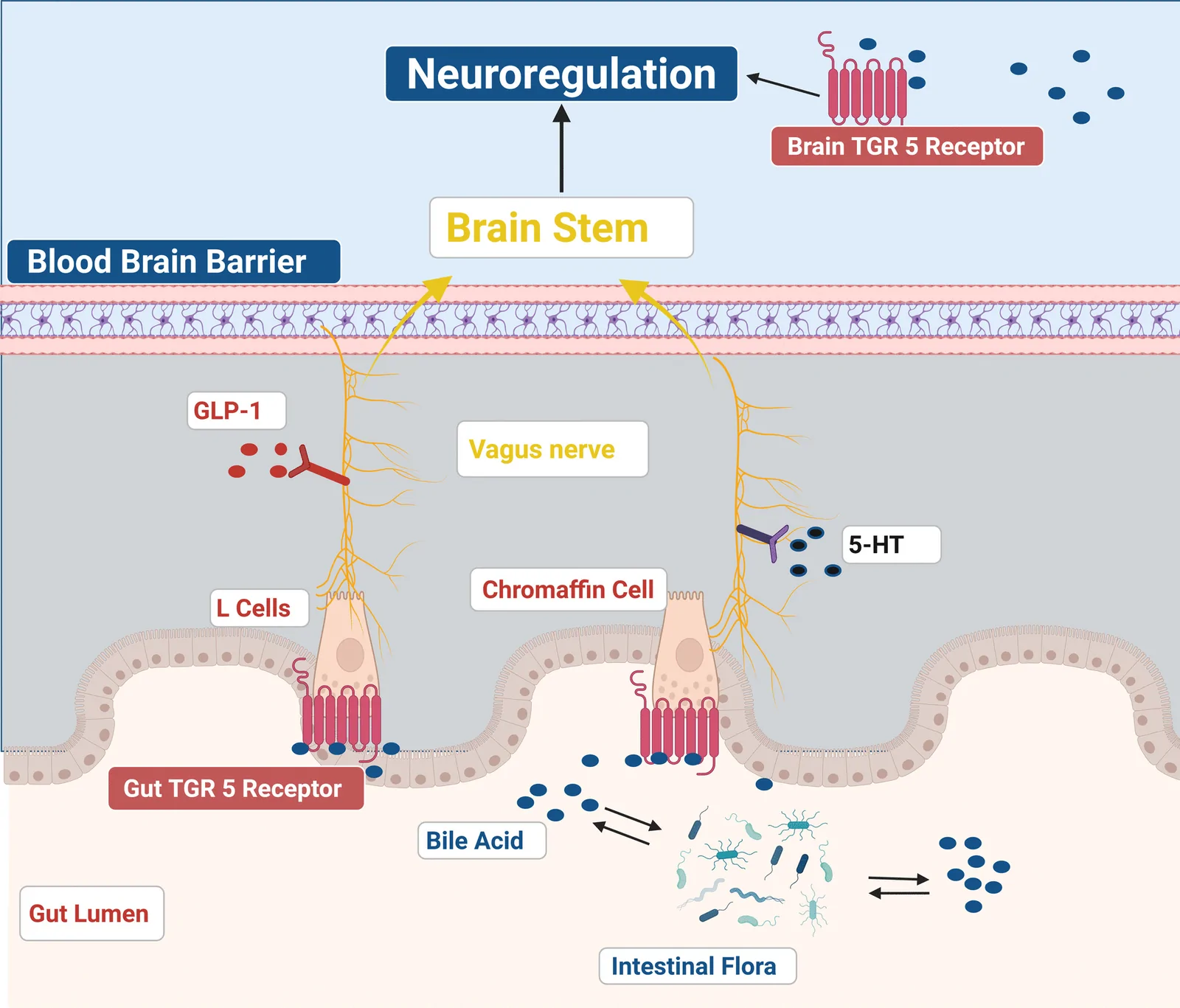
Gut–brain neuroregulatory signaling mediated by bile acid–TGR5 activation and vagal afferent pathways. Bile acids (BAs) generated in the gut lumen and shaped by the intestinal flora activate TGR5 receptors expressed on intestinal epithelial chemosensory cells, including enteroendocrine L cells and chromaffin cells. TGR5 stimulation promotes the release of key gut-derived neuromodulators such as glucagon-like peptide 1 (GLP-1) and serotonin (5-HT), which signal locally to vagal afferent fibers. These vagal inputs relay visceral information to the brainstem, initiating central circuits involved in neuroregulation. In addition, circulating or locally produced bile acids may cross the blood–brain barrier (BBB) and directly activate brain TGR5 receptors, further integrating peripheral metabolic and microbial cues with central autonomic and behavioral control. Overall, the figure highlights a bile acid–dependent gut–brain communication axis that links microbial metabolic signaling to brainstem-driven neuromodulatory outcomes

Taken together, bile acids that cross the BBB have a direct neuroprotective effect against the pathogenesis of AD. In addition, bile acids modulate gut microbiota, the TGR5/FXR axis, and gut 5-HT, thereby improving the gut–brain axis and, indirectly, protecting against AD neuropathology (Fig. 8).

However, the present review has several limitations, including the scarcity of clinical studies, which limits the clinical significance of bile acids in AD. Additionally, the causal relationship between bile acids in AD was not explored at a molecular level. Furthermore, the use and long-term effects of bile acid analogs or receptor activators in AD patients were not revised accordingly. Despite these limitations, this review suggests that bile acids, by modulating brain signaling pathways, may offer clinical benefits in the management of AD, a claim that warrants further study to confirm. The indirect effects of bile acids on AD neuropathology are summarized in Table 4.

**Table 4:** Indirect effects of bile acids on AD neuropathology

Studies	Findings	Ref
Clinical	Case–control studies have shown that microbial dysbiosis alters lipid metabolism, including upregulation of bacterial species responsible for the synthesis of hydrophobic secondary bile acids in patients with PD	(Shibuya et al. 2024)
Preclinical	Secondary bile acids and toxic metabolites arising from gut dysbiosis can affect vagal neural activity, which is impaired in AD	(Siddeeque et al. 2024; Singh and Aran 2026)
Preclinical	The bile acids trigger GLP-1 release from intestinal L cells, which has a neuroprotective effect against AD, by activating TGR5 and FXR and increasing ASBT expression in mice	(Vargas-Caballero et al. 2022)
Preclinical	Bile acids may improve cognitive and metabolic functions by stimulating GLP-1 release via TGR5 and modulating gut–brain signaling pathways	(Wang et al. 2021)
Preclinical	The bile acids, by activating FGF19 release from the intestine, trigger hypothalamic FGFR and enhance the neuroprotective release of NPY, which regulates brain insulin sensitivity	(Ye et al. 2024)
Preclinical	Bile acids, by modulating TGR5/FXR, increase gut 5-HT, which in turn attenuates the development and progression of AD via the gut–brain axis	(Zuo et al. 2019)

## Conclusions

Bile acids have emerged as biologically active signaling molecules that extend far beyond their classical functions in digestion and hepatic metabolism. Current evidence supports a key role for bile acid–driven pathways in shaping systemic inflammation, glucose regulation, and neuroimmune communication via the liver–gut–brain axis, processes increasingly recognized as central contributors to AD pathophysiology. Importantly, bile acid signaling appears to be context-dependent, influenced by bile acid species composition, microbiota-derived biotransformations, receptor specificity (notably FXR and TGR5), and disease stage. Dysregulation of bile acid homeostasis may exacerbate AD-related mechanisms, including oxidative stress, BBB impairment, microglial activation, synaptic dysfunction, and tau-associated neurodegeneration. In contrast, specific hydrophilic bile acids and receptor-mediated pathways may have neuroprotective and anti-inflammatory effects.

Despite growing mechanistic and clinical interest, critical knowledge gaps remain regarding causal relationships, optimal therapeutic targeting, and patient-specific bile acid signatures that could guide precision medicine approaches. Future research should prioritize standardized bile acid profiling in longitudinal cohorts, integration of metabolomics with microbiome and neuroimaging measures, and controlled experimental studies to define receptor-specific effects across neural and glial cell populations. Collectively, these insights position bile acid signaling as a promising and underexplored frontier in AD research, with potential applications in biomarker development and the design of novel pharmacological and microbiome-directed therapeutic strategies.

## Data Availability

This manuscript does not report data generation or analysis.
